# Heart Rate Variability Biofeedback Intervention for Reduction of Psychological Stress During the Early Postpartum Period

**DOI:** 10.1007/s10484-014-9259-4

**Published:** 2014-09-20

**Authors:** Naoko Kudo, Hitomi Shinohara, Hideya Kodama

**Affiliations:** Department of Maternity Child Nursing, School of Health Science, Akita Graduate School of Medicine and Faculty of Medicine, 1-1-1 Hondo, Akita-shi, 010-8543 Japan

**Keywords:** Heart rate variability, Biofeedback, Edinburgh Postnatal Depression Scale, Early postpartum period, Psychological stress

## Abstract

This study examined the effectiveness of heart rate variability (HRV) biofeedback intervention for reduction of psychological stress in women in the early postpartum period. On postpartum day 4, 55 healthy subjects received a brief explanation about HRV biofeedback using a portable device. Among them, 25 mothers who agreed to implement HRV biofeedback at home were grouped as the biofeedback group, and other 30 mothers were grouped as the control group. At 1 month postpartum, there was a significant decrease in total Edinburgh Postnatal Depression Scale score (*P* < 0.001) in the biofeedback group; this change was brought about mainly by decreases in items related to anxiety or difficulty sleeping. There was also a significant increase in standard deviation of the normal heartbeat interval (*P* < 0.01) of the resting HRV measures in the biofeedback group after adjusting for potential covariates. In conclusion, postpartum women who implemented HRV biofeedback after delivery were relatively free from anxiety and complained less of difficulties sleeping at 1 month postpartum. Although the positive effects of HRV biofeedback may be partly attributable to intervention effects, due to its clinical outcome, HRV biofeedback appears to be recommendable for many postpartum women as a feasible health-promoting measure after childbirth.

## Introduction

Immediately after delivery, mothers are required to adapt to a new lifestyle that focuses on childcare. Many mothers find the process of meeting the demands of their new lifestyle a joyful experience. However, some mothers have trouble getting used to the new routines and responsibilities and experience high stress levels. The early postpartum period is a critical time during which women have an increased risk for depression (Cox et al. 1993; Ross and Dennis 2009; Klainin and Arthur 2009). Therefore, effective interventions that help these women transition through this stressful period should be available.

The autonomic nervous system plays an important role in human stress reactions. During usual stress reactions, the introduction of a stressor activates the sympathetic nervous system; the system returns to its former state when the stress fades. When subjects are exposed to chronic stress beyond the range where physiological functions are reversible, their everyday autonomic balance shifts toward a sympathetic-predominant state as a result of parasympathetic withdrawal. However, this persistent attenuation of parasympathetic activity may deteriorate the regulatory capability of physiological functions for external stressors (Porges 1995; McEwen 2004; Thayer and Sternberg 2006). In late pregnancy, the balance of the autonomic nervous system of the resting period is shifted toward a sympathetic-predominant state with parasympathetic withdrawal, probably due to adaptive responses against hemodynamic changes and aortocaval compression caused by the enlarged uterus (Kuo et al. 2000; Walther et al. 2005; Matsuo et al. 2007). After delivery, this specific condition rapidly returns to a non-pregnant state, and the recovery process includes parasympathetic activation. If this recovery process does not proceed normally, that is, if sympathetic-predominant autonomic balance is not smoothly recovered, postpartum women became more vulnerable to external stressors and may develop physical and/or psychiatric disorders.

Heart rate variability (HRV) biofeedback is a training method to control one’s breathing to the resonate frequency of about five to six breaths per minute, at which the amplitude of HRV is maximized; this may strengthen the baroreflex, thus improving autonomic functioning (Lehrer et al. 2003; Vaschillo et al. 2006). HRV biofeedback has been shown to contribute to the treatment of a variety of diseases with autonomic dysfunctions, including stress-related psychiatric disorders (Karavidas et al. 2007; Reiner 2008; Siepmann et al. 2008; Zucker et al. 2009; Weber et al. 2010; Tan et al. 2011; Beckham et al. 2013) or stress-related chronic pain (Hassett et al. 2007; Hallman et al. 2011). Furthermore, HRV biofeedback may be available as a stress management method for healthy subjects under relatively stressful conditions (Henriques et al. 2011; Ratanasiripong et al. 2012; Whited et al. 2014). Theoretically, HRV biofeedback is beneficial in most mothers whose autonomic balance tends to shift toward a sympathetic-predominant state. Some portable devices for HRV biofeedback are marketed worldwide (Ebben et al. 2009), and HRV biofeedback is a feasible intervention during the early postpartum period. However, it remains questionable whether HRV biofeedback results in favorable modifications in autonomic functioning of healthy subjects (Lehrer and Eddie 2013), and effectiveness of HRV biofeedback in healthy postpartum women should be carefully verified before recommending it to mothers as a health-promoting measure after childbirth.

The objective of the present study was to examine the effectiveness of HRV biofeedback intervention for reduction of psychological stress in women in the early postpartum period. We investigated whether implementation of HRV biofeedback for 4 weeks immediately after delivery could contribute to reduction of the Edinburgh Postnatal Depression Scale (EPDS), a standardized self-reported questionnaire to identify women who have postpartum depression (Cox et al. 1987). The EPDS has been shown to be able to detect perinatal anxiety disorders as well (Matthey 2008; Matthey et al. 2013). Additionally, resting HRV measures in each woman were evaluated as indicators of a fundamental autonomic neural state, and impacts of HRV biofeedback on the measures were assessed. Our hypothesis was that implementation of HRV biofeedback immediately after delivery would result in lower scores on the EPDS and increased HRV measures at 1 month postpartum and that there would be close correlations between EPDS and HRV measures.

## Methods

### Study Subjects

The study protocol was approved by the Ethics Committee of Akita University Graduate School of Medicine and the Faculty of Medicine. Subjects were recruited from mothers who gave birth at Akita University Hospital between October 1, 2011 and September 30, 2013; recruitment took place 4 days postpartum. Only healthy mothers who had experienced vaginal deliveries of a single infant, without any medical complications, were included. Mothers who habitually drank alcohol or smoked were excluded. Written informed consent was obtained from mothers who agreed to participate in the study.

On postpartum day 4, subjects completed a questionnaire detailing demographic data, including age, gestational age, parity, height, and employment status. As a part of a routine health checkup, body weight, blood pressure, heart rate, and body temperature were measured. Around 4 days after birth, mothers often experience a transient mental disorder called maternity blues. The Stein scale for maternity blues (Stein 1980) was used to determine whether subjects suffered from this condition.

### Heart Rate Variability Biofeedback

All subjects received a brief explanation about HRV biofeedback on postpartum day 4. If subjects agreed to use HRV biofeedback at home, detailed directions regarding how to implement HRV biofeedback using a portable device (StressEraser, Helicor, Inc., New York, NY, USA) were provided. This device records blood vessel pulse waves in the index finger in real time and displays HRV as a waveform on the screen. When users synchronize the rhythm of their breathing with this waveform, they create a resonance between breathing-induced HRV and HRV due to Mayer waves from arterial pressure. When a resonance is completely established, their HRV becomes maximized, and parasympathetic tone is enhanced. The degree of consistency between the HRV waveform on the screen and breathing rhythm is shown on the screen in real time above each individual waveform as a point display ranging from 1 to 3, with 3 points representing the best synchronization.

Subjects who agreed to implement HRV biofeedback learned to use the device while they were in the hospital and took the device home about 6 days after delivery. According to instructions for the device, subjects were recommended to undergo HRV biofeedback daily with a score of 30 points or more per session and with a sufficient number of sessions a day to achieve a total score of 100 points or more. They were also asked to record their performance daily on a provided chart. Subjects took part in a telephone interview around 2 weeks after discharge to check their compliance with HRV biofeedback. After 4 weeks, subjects visited our hospital for a routine 1-month postnatal check-up. Subjects who did not agree to use biofeedback served as the control group.

### Heart Rate Variability Analysis

The resting HRV of all subjects was recorded on day 4 and 1 month postpartum by photoplethysmography (Heart Rhythm Scanner, Biocom Technologies, Poulsbo, WA, USA). Data were collected between 10:00 am and noon, after subjects had confirmed that they had not eaten, drank, or smoked during the previous 2 h. Subjects were instructed to rest in the supine position for 5–10 min in a quiet room and breathe slowly. Next, the heart rate scanner optical ear clip sensor was attached to the pinna of the ear. Pulse intervals were recorded for 5 min, during which participants were requested to remain in the supine position. Data were immediately uploaded to a personal computer and HRV measures were calculated. The HRV measures of interest included the standard deviation of the normal heartbeat interval (SDNN), the high-frequency (HF) power in the 0.15–0.4 Hz waveband, and the low-frequency (LF) power in the 0.04–0.15 Hz wave band (Task Force 1996).

### Edinburgh Postnatal Depression Scale

On day 4 postpartum and at the 1-month postpartum check-up at our hospital, mental state was assessed in all subjects using the EPDS. The EPDS is a 10-item self-rating questionnaire developed to detect probable depression in the first 8 weeks after childbirth (Cox et al. 1987) and appears to detect perinatal anxiety disorders as well (Matthey 2008; Matthey et al. 2013). Each item is scored on a scale of 0–3, and the total score ranges from 0 to 30. A score ≥10 points indicates a high risk for postpartum depression.

Each woman completed the EPDS by herself on day 4 postpartum, but the EPDS at the 1-month postpartum was evaluated during a face-to face-interview with a clinical psychologist who had no direct connection to this study; all interviews took place in a private room. Because our hospital has a rule requiring that all mothers be asked to undergo an interview with a clinical psychologist 1 month after giving birth, the subjects of this study were unaware that the EPDS was being used as the study scale when they were interviewed. After interview, we obtained informed consent from each subject to use this score at the 1-month postpartum for outcome measures in this study.

### Statistics

Statistical analyses were performed using the Statistical Package for the Biosciences (Nankodo, Tokyo, Japan) or IBM SPSS Statistics (version 21.0 Static Base and Advanced Statistics, IBM Japan, Tokyo, Japan). Because the distributions for HF power and LF power (the frequency domain analysis values for HRV) approached a normal distribution, logarithmic conversion was performed before analysis. Intergroup comparisons and correlations were analyzed by parametric or nonparametric methods depending on whether or not data were normally distributed. Two-way factorial analysis of variance was used to compare the repeated HRV measures between mothers who underwent biofeedback with those who did not. Group differences of HRV measures at 1 month postpartum were examined by analysis of covariance, adjusted for maternal age, parity, systolic blood pressure, and body mass index. Data were expressed as mean ± SD, with *P* < 0.05 regarded as statistically significant.

## Results

Fifty-five mothers were approved to participate in this study. Among them, 25 mothers who agreed to implement HRV biofeedback were grouped as the biofeedback group, and 30 mothers who did not want to use HRV biofeedback were grouped as the control group. Table 1 presents comparisons of demographic factors, physical findings, and HRV measures on postpartum day 4 between groups. There were significant differences between groups in terms of parity, gestational age, and systolic blood pressure. The proportion of primiparous mothers was significantly higher in the biofeedback group. Maternity blues was diagnosed in 16 mothers (29.1 %), and the proportion of affected mothers was comparable between groups. There were no significant differences in HRV measures or EPDS between groups on postpartum day 4.

**Table 1:** Comparisons of demographic factors, physical findings, heart rate variability measures, and Edinburgh postnatal depression scale on postnatal day 4 between the biofeedback and the control groups

	Biofeedback group n = 25	Control group n = 30	*P* ^a^
*Demographic factors*			
Age (years)	30.5 ± 5.7	33.4 ± 6.6	0.086
Primiparous (number, %)	22 (88.9)	19 (65.3)	0.032
Gestational age (weeks)	39.1 ± 1.0	38.5 ± 0.8	0.030
*Physical findings*			
Body mass index (kg/m^2^)	23.5 ± 3.1	24.1 ± 3.0	0.412
Systolic blood pressure (mmHg)	109 ± 10	116 ± 12	0.023
Maternity blues	9 (38.9)	7 (23.1)	0.303
*Heart rate variability measures*			
Heart rate (beats/min)	74.3 ± 7.5	77.1 ± 7.6	0.186
SDNN (ms)	38.7 ± 11.5	40.0 ± 16.8	0.743
HF power (log, ms^2^)	4.46 ± 0.94	4.69 ± 0.84	0.358
LF power (log, ms^2^)	4.59 ± 0.98	4.70 ± 1.00	0.684
*Edinburgh postnatal depression scale*			
Total score	4.60 ± 1.99	4.20 ± 2.08	0.317^b^

Values are mean ± SD (range) or numbers (%)

*SDNN* standard deviation of normal-to-normal beat intervals, *LF* low frequency, *HF* high frequency

^a^Group differences were examined by student *t* test or chi-square test

^b^Group differences were examined by Wilcoxon test

According to daily charts from mothers, all mothers in the biofeedback group implemented at least one session of HRV biofeedback every day, and 20 mothers (80 %) achieved a total of 100 points in all sessions. Four mothers reported that they could not achieve 100 points on about 2–5 days because they fell asleep while implementing HRV biofeedback before a score reached that point. One multiparous mother implemented HRV biofeedback and achieved less than 100 points on most days because the laborious care required for her older child.

**Fig. 1:**
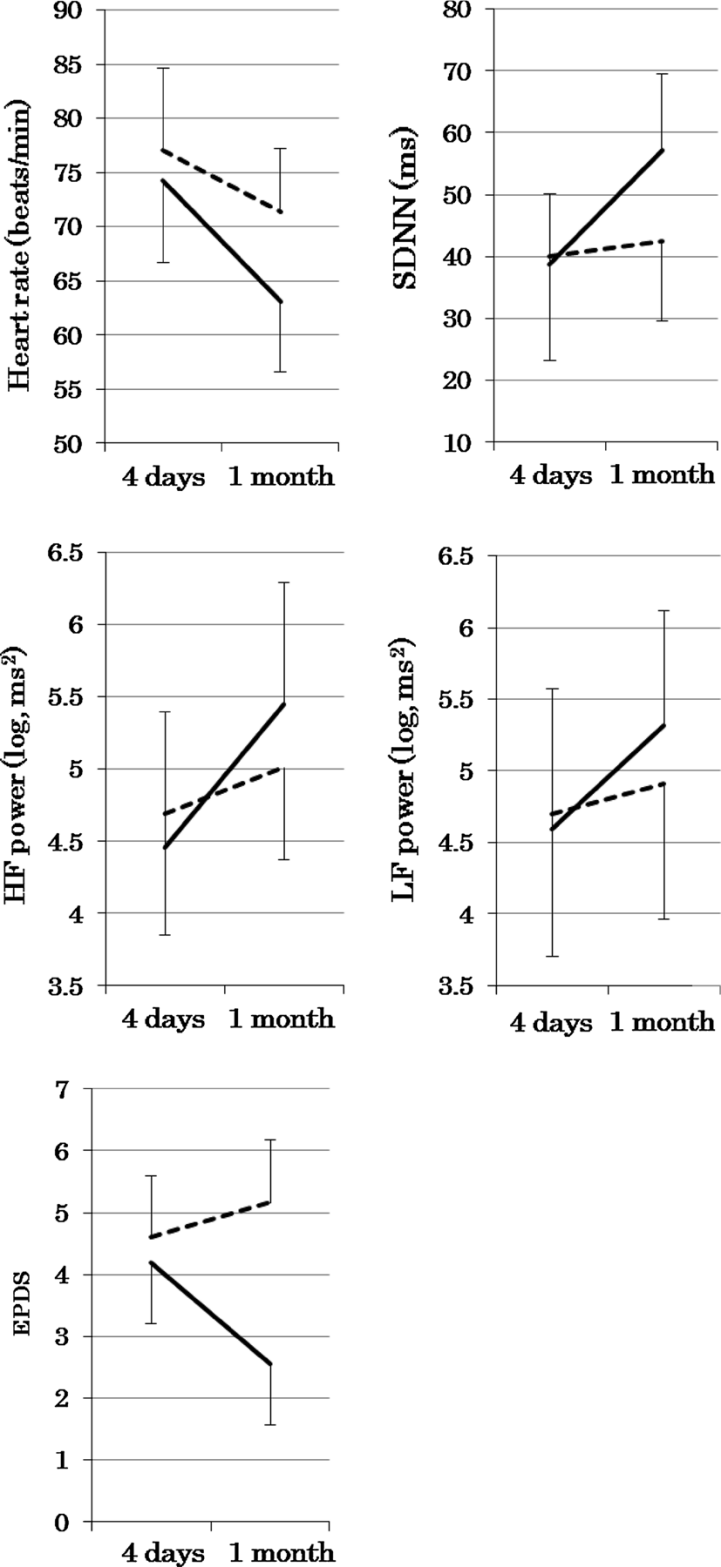
Mean ± SD of each HRV measure or Edinburgh Postnatal Depression Scale from 4 days to 1 month postpartum in the biofeedback (*solid lines*) and control (*dash lines*) groups. Significant interactive differences (group × time) were found for heart rate, SDNN, and HF power (see Table 2). *SDNN* standard deviation of normal-to-normal beat intervals, *LF* low frequency, *HF* high frequency, *EPDS* Edinburgh Postnatal Depression Scale

Table 2 presents comparisons of HRV measures or EPDS between groups from 4 days to 1 month postpartum. All measures exhibited significant time-dependent changes from 4 days to 1 month postpartum, including a decrease in heart rate, increases in other HRV measures, and a decrease in EPDS. Significant interactive differences that (group × time) change between groups were found for heart rate, SDNN, HF power, and EPDS, indicating that these measures changed in different ways between two groups. Figure 1 presents changes of mean values of each HRV measure or EPDS from 4 days to 4 weeks postpartum in the two groups. The magnitude of changes in heart rate, SDNN, and HF power appeared to be greater in the biofeedback group, as compared to those in the control group. The time-course decrease in EPDS was observed in the biofeedback group only.

**Table 2:** Comparisons of heart rate variability measures or Edinburgh postnatal depression scale from 4 days to 1 month postpartum between the biofeedback and control groups

	4 days Mean ± SD	1 month Mean ± SD	Time^a^		Time × Group^a^		Group^a^	
			F_(1,53)_	*P*	F_(1,53)_	*P*	F_(1,53)_	*P*
*Heart rate (beats/min)*								
Biofeedback	74.3 ± 7.5	63.1 ± 5.8	78.34	<0.001	8.30	0.006	11.67	0.198
Control	77.1 ± 7.6	71.4 ± 6.5						
*SDNN (ms)*								
Biofeedback	38.7 ± 11.5	57.2 ± 12.3	21.65	<0.001	12.41	0.001	5.26	0.090
Control	40.0 ± 16.8	42.5 ± 12.8						
*HF power (log, ms* ^*2*^ *)*								
Biofeedback	4.46 ± 0.94	5.45 ± 0.84	19.17	<0.001	4.89	0.031	0.46	0.501
Control	4.69 ± 0.84	5.01 ± 0.64						
*LF power (log, ms* ^*2*^ *)*								
Biofeedback	4.59 ± 0.98	5.32 ± 0.80	11.00	0.002	3.46	0.068	0.51	0.477
Control	4.70 ± 1.00	4.91 ± 0.95						
*Edinburgh Postnatal Depression Scale*								
Biofeedback	4.20 ± 2.08	2.56 ± 2.26	4.60	0.037	19.43	<0.001	7.68	0.008
Control	4.60 ± 1.99	5.17 ± 2.45						

Values are mean ± SD

*SDNN* standard deviation of normal-to-normal beat intervals, *LF* low frequency, *HF* high frequency

^a^Values are F(*P*) of two-way (time × group) repeated measures ANOVA

**Fig. 2:**
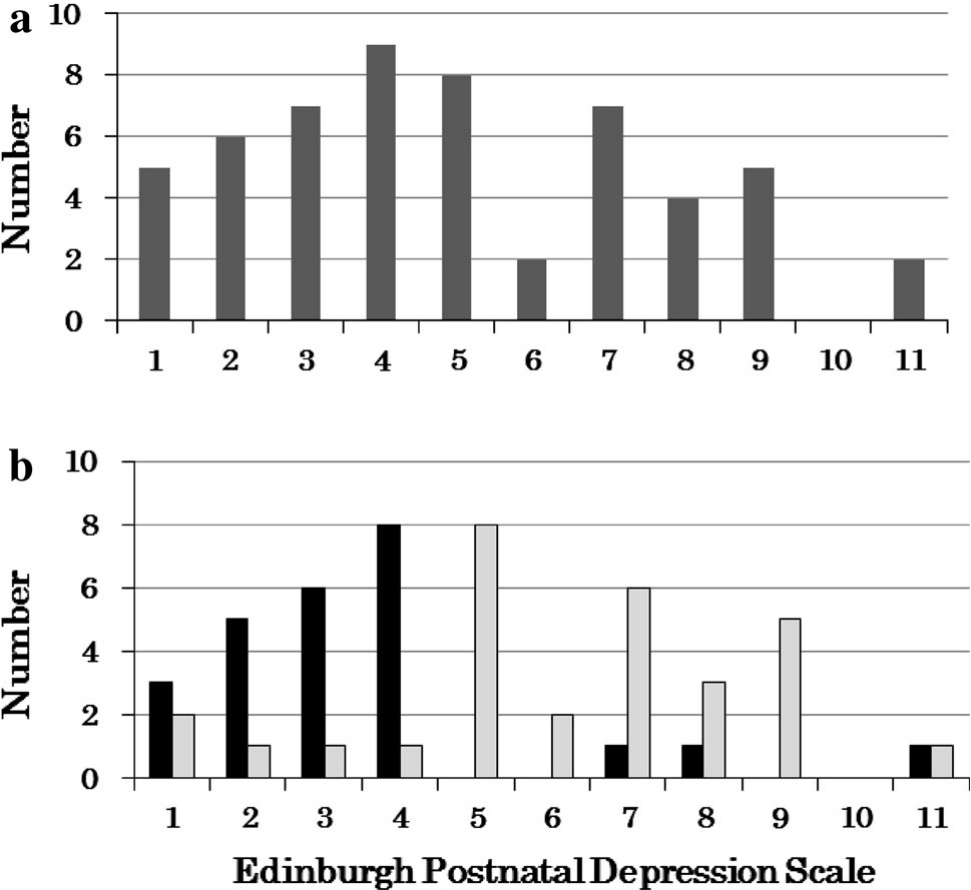
Distribution of Edinburgh Postnatal Depression Scale in women at 1 month postpartum. Distributions of all women (n = 55) are presented in (**a**), and distributions of women in each group (*black bars* for biofeedback, n = 25 and *gray bars* for control, n = 30) are presented in (**b**)

Figure 2 presents distributions of EPDS in women at one month postpartum. Distributions of all women (n = 55) are presented in Fig. 2a, and only two women scored ≥10, indicating a high risk for postnatal depression. Distributions of women in each group are separately presented in Fig. 2b. In the biofeedback group, 22 of 25 mothers (88.0 %) presented the EPDS of below 5, and, in the control group, 25 of 30 mothers (83.3 %) presented the EPDS of above 4.

Table 3 presents comparisons of HRV measures and EPDS (each item and total score) at 1 month postpartum between groups. In terms of HRV measures after adjusting for maternal age, parity, systolic blood pressure, and body mass index, there were significant decreases in heart rate and increases in SDNN in the biofeedback group compared with the control group. There were significant differences between groups in total EPDS score (*P* < 0.001, Wilcoxon test). Among the EPDS items, significant differences between groups were found in three items related to anxiety (items 3–5), one item related to difficulty sleeping (item 7) and one item related to sad and miserable feelings (item 8). Only one woman in the biofeedback group and one woman in the control group scored ≥10, indicating that they were at high risk for postnatal depression.

**Table 3:** Comparisons of heart rate variability measures and Edinburgh Postnatal Depression Scale (each item and total score) at 1 month postpartum between the biofeedback and the control groups

	Biofeedback group n = 25	Control group n = 30	*P*
*Heart rate variability measures* ^*a*^			
Heart rate (beats/min)	63.3 ± 5.4	71.2 ± 6.1	<0.001
SDNN (ms)	54.7 ± 10.7	44.6 ± 10.1	0.002
HF power (log, ms^2^)	5.38 ± 0.78	5.07 ± 0.48	0.110
LF power (log, ms^2^)	5.26 ± 0.78	4.96 ± 0.76	0.196
*Edinburgh Postnatal Depression Scale* ^*b*^			
1. I have been able to laugh and see the funny side of things	0.04 ± 0.20	0.00 ± 0.00	0.290
2. I have looked forward to things with enjoyment	0.04 ± 0.20	0.10 ± 0.31	0.409
3. I have blamed myself unnecessarily when things went wrong	0.60 ± 0.50	1.23 ± 0.63	<0.001
4. I have been anxious or worried for no good reason	0.60 ± 0.65	1.00 ± 0.74	0.040
5. I have felt scared or panicky for no good reason	0.08 ± 0.11	0.60 ± 0.68	0.001
6. Things have been getting on top of me	0.96 ± 0.54	1.27 ± 0.58	0.050
7. I have been so unhappy that I have had difficulty sleeping	0.08 ± 0.28	0.37 ± 0.49	0.014
8. I have felt sad or miserable	008 ± 0.28	0.37 ± 0.49	0.014
9. I have been so unhappy that I have been crying	0.08 ± 0.28	0.13 ± 0.35	0.542
10. The thought of harming myself has occurred to me	0.00 ± 0.00	0.10 ± 0.31	0.112
Total score	2.56 ± 2.26	5.17 ± 2.45	<0.001

*SDNN* standard deviation of normal-to-normal beat intervals, *LF* low frequency, *HF* high frequency

^a^Group differences were examined by analysis of covariance, adjusted for maternal age, parity, systolic blood pressure, and body mass index

^b^Group differences were examined by Wilcoxon test

Table 4 presents the correlation coefficients of total EPDS with each HRV measure at 1 month postpartum in all mothers (n = 55). The EPDS score exhibited a significant positive correlation with heart rate, and significant negative correlations with SDNN and HF power (Spearman’s rank correlation test).

**Table 4:** Correlation coefficients of total Edinburgh Postnatal Depression Scale with each heart rate variable measure in all mothers at 1 month postpartum

	R^a^	*P*
Heart rate (beats/min)	0.476	<0.001
SDNN (ms)	−0.277	0.04
HF power (log, ms^2^)	−0.357	0.008
LF power (log, ms^2^)	−0.211	0.122

*SDNN* standard deviation of normal-to-normal beat intervals, *LF* low frequency, *HF* high frequency

^a^Spearman’s rank correlation coefficient

## Discussion

EPDS scores at 1 month postpartum were significantly lower in the biofeedback group than in the control group, suggesting that use of HRV biofeedback after delivery contributed to reduction of psychological stress in postpartum women. Comparisons of each item in the EPDS between groups showed that HRV biofeedback contributed to alleviation of items related to anxiety (Matthey 2008) and difficulty sleeping. Anxiety is probably among the most common negative emotions for postpartum women. In a community sample of 8,323 pregnant women, approximately 15 % of women reported elevated anxiety in the antenatal period, and rates were comparable in the postnatal period (Heron et al. 2004). In fact, anxiety disorders are more common than depressive disorders in the perinatal period (Matthey et al. 2013). Therefore, reduction of anxiety symptoms with HRV biofeedback, which was reported by other studies (Reiner 2008; Henriques et al. 2011; Ratanasiripong et al. 2012), may be particularly beneficial for postpartum women. HRV biofeedback was reported to shorten sleep latency (Ebben et al. 2009), prolong deeper sleep stages (Sakakibara et al. 2013), and ameliorate insomnia (McLay and Spira 2009). Due to childcare responsibilities, postpartum women sleep less during the early weeks following delivery than during pregnancy and other periods of the reproductive age (Lee et al. 2000). These impaired sleep patterns are strongly correlated with depressive symptoms in postpartum women (Dørheim et al. 2009). Therefore, it is likely that sleep-promoting effects of HRV biofeedback also contribute to reduction of psychological stress in some postpartum women.

From 4 days to 1 month postpartum, there were significant reductions in heart rate and elevations in SDNN, HF power, and LF power. HF power is an established index of cardiac vagal tone, reflecting respiratory sinus arrhythmia. Although LF power was previously thought to reflect cardiac sympathetic outflow, several researchers believe that the HRV power spectrum, including the LF component, is mainly determined by the parasympathetic system (Grassi and Esler 1999; Reyes del Paso et al. 2013). Therefore, we regarded that increases in both LF and HF power would reflect increased parasympathetic activity after delivery. The magnitude of changes in heart rate, SDNN, and HF power were larger in the biofeedback group, and thus, HRV biofeedback may exaggerate parasympathetic activation during the early postpartum period. However, this difference may also be attributable to the fact that the demographics of subjects were biased. The biofeedback group included a larger proportion of primiparous mothers with relatively younger ages. The activation of parasympathetic tone after delivery may be more evident among younger, primiparous mothers. Therefore, we should attach more importance to results showing significant group differences in heart rate and SDNN at 1 month postpartum after controlling for the influence of covariates, including maternal age and parity.

There were significant positive correlations between EPDS and heart rate, and negative correlations between EPDS and both SDNN and HF powers. In general, low HRV is thought to indicate decreased parasympathetic activity. Therefore, a significant increase in resting SDNN, the index of overall HRV, may indicate that use of HRV biofeedback resulted in increased parasympathetic tone in the resting state. However, the meaning attached to increases in SDNN without increases in HF and/or LF power in the present study should be carefully considered because SDNN simply increases when heart rate decreases, as was observed in the present study. Previously, several studies analyzed HRV measures as an outcome of HRV biofeedback, and significant increases in SDNN or LF power during HRV biofeedback were constantly reported (Lehrer et al. 2003; Karavidas et al. 2007; Hassett et al. 2007). However, although several studies investigated whether there were carry-over effects of HRV biofeedback on resting HRV, conclusions were inconsistent. Some studies demonstrated positive impacts of HRV biofeedback on resting HRV measures, such as SDNN (Zucker et al. 2009; Del Pozo et al. 2004) or LF power (Hallman et al. 2011), whereas other studies reported that the influences were rare or nonexistent (Lehrer et al. 2003; Karavidas et al. 2007; Siepmann et al. 2008; Swanson et al. 2009; Henriques et al. 2011). Therefore, it may be true that positive effects of HRV biofeedback cannot be clearly explained by changes in daily autonomic functioning. Wheat and Larkin (2010) stated that, because clinical and physiological outcome do not improve concurrently, the mechanism by which HRV biofeedback results in salutary effects is still unclear.

Compliance with HRV biofeedback was high among women in this study, with as many as 20 mothers (80 %) achieving a total of 100 points or more in all sessions. Clinical outcomes of the biofeedback group were favorable, although it was probable that these effects resulted, in part, from the intervention effects. In our experience, HRV biofeedback serves as a useful communication tool between medical staff and mothers, as we noted that investigators and some mothers using HRV biofeedback achieved a closer relationship throughout this study. In another study, dizziness occurred in 15 % of 24 patients with anxiety disorders who used HRV biofeedback, a side effect that may have resulted from hyperventilation (Reiner 2008). No mothers in this study complained about this symptom. Therefore, HRV biofeedback is a feasible, effective, and safe intervention for most postpartum women. Thus, if staff members recommend HRV biofeedback with enthusiasm, a considerable number of mothers may be willing to use this treatment. However, it remains questionable whether HRV biofeedback is really advantageous to healthy users. Lehrer and Eddie (2013) stated that HRV biofeedback enhanced the negative feedback loop, including the baroreflex, but this might weaken reflexes dependent on oscillations at other frequencies. This raises the concern that frequent, long-term use of HRV biofeedback may weaken adaptability of the physical control system to external stressors. Therefore, for postpartum women, it may be preferable to implement HRV biofeedback for a relatively short period daily (about 20 min, as recommended by Lehrer and Eddie 2013), and to limit the period of HRV biofeedback to the first month after delivery, when stress is most likely to occur.

Several limitations of the present study warrant discussion. First, this was not a random study and demographics were biased, although use of appropriate statistical analyses was able to control for potential covariates to some extent. Second, the resting HRV was recorded by photoplethysmography, not by electrocardiography, and thus, accuracy of HRV measures was less than ideal. Third, subjective influences may have occurred in the biofeedback group when they answered questions from the EPDS interviewer, and the decrease in EPDS in the biofeedback group may be largely due to intervention effects. The absence of an active control made it difficult to validate genuine effects of HRV biofeedback. Forth, evaluation of individual stress levels relying on single EPDS may be incorrect in some women, and introduction of multilateral evaluation (e.g., simultaneous estimation of another stress scale or using biochemical markers) may have provide more accurate information on stress levels in postpartum women. Fifth, our results do not indicate that HRV biofeedback contributes to a reduction in the risk of postpartum depression. Our study subjects included only two women who scored ≥10 on the EPDS, indicating a high risk for postnatal depression. There is no evidence that a difference in scores that fall within the normal range reflects a difference in the actual risk for postpartum depression.

In conclusion, results in this study partially supported our hypothesis that implementation of HRV biofeedback immediately after delivery resulted in lower EPDS scores and increased HRV measures at 1 month postpartum. The mothers who used HRV biofeedback were relatively free from anxiety and complained less of difficulties sleeping; however, the lack of a random study design and an active control group means that these findings should be interpreted with caution. HRV biofeedback intervention was found to reduce heart rate and increase SDNN in the resting period, but increases in SDNN without increases in HF or LF powers provide inconclusive evidence of parasympathetic activations. However, due to its clinical effectiveness and feasibility, HRV biofeedback appears to be recommendable for many postpartum women after childbirth, especially when they are worried about upcoming changes in routines and the responsibilities of childcare.
